# Supplementary material for: Structural insights into trans-histone regulation of H3K4 methylation by unique histone H4 binding of MLL3/4
Source: Nat Commun. 2019 Jan 3;10:36. doi: 10.1038/s41467-018-07906-3 (PMC6318328; doi:10.1038/s41467-018-07906-3)
Supplement: Supplementary file 1 — Supplementary Information [file 41467_2018_7906_MOESM1_ESM.pdf]

Supplementary Information

Structural insights into trans-histone regulation of H3K4 methylation by unique histone H4 binding of MLL3/4

Liu, et al.

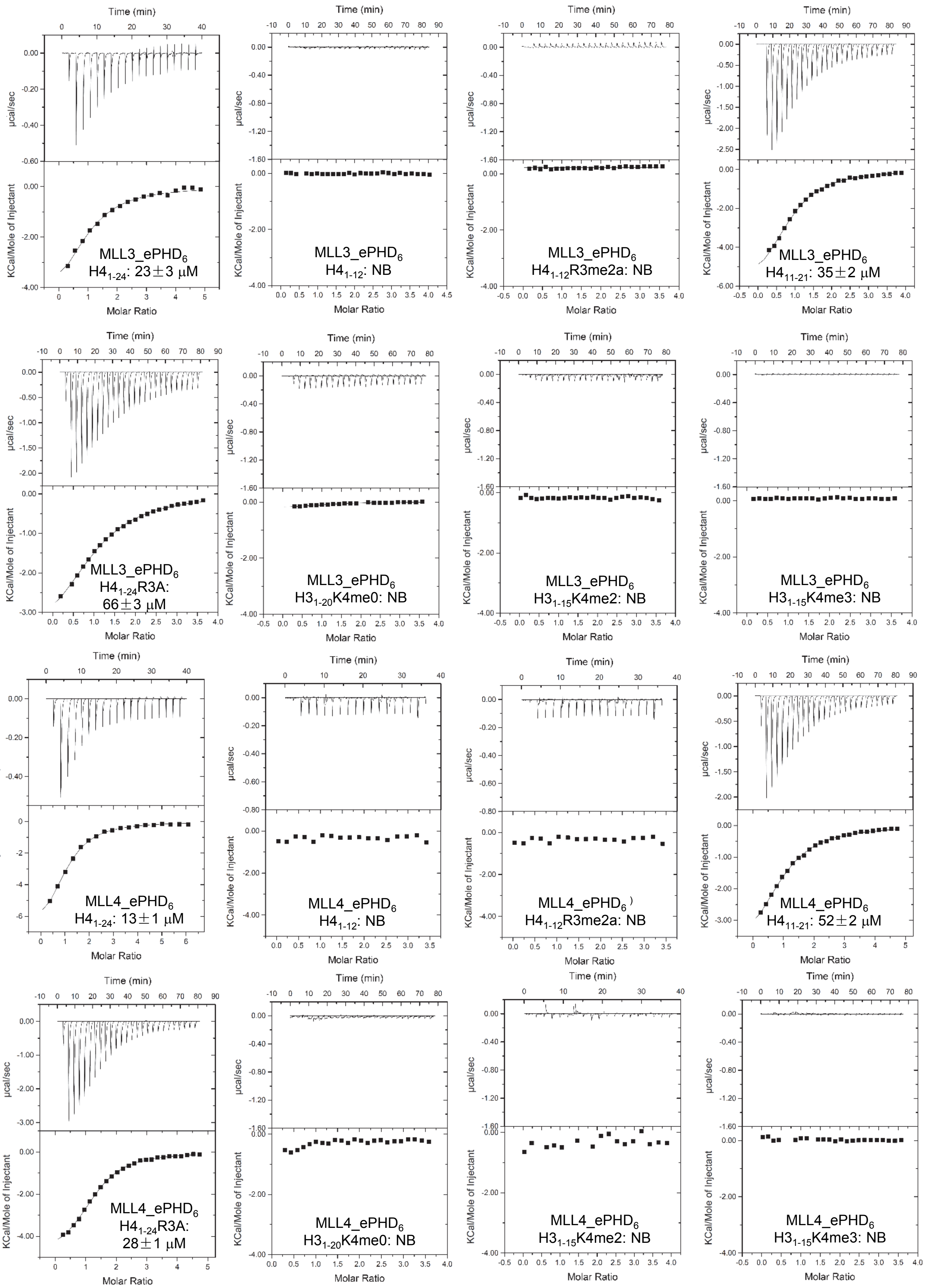

Supplementary Fig. 1 ITC titration curves for different histone peptides to the extended PHD$_6$ domains (ePHD$_6$) of MLL3 and MLL4. NB: no detectable binding. Related to Table 1.

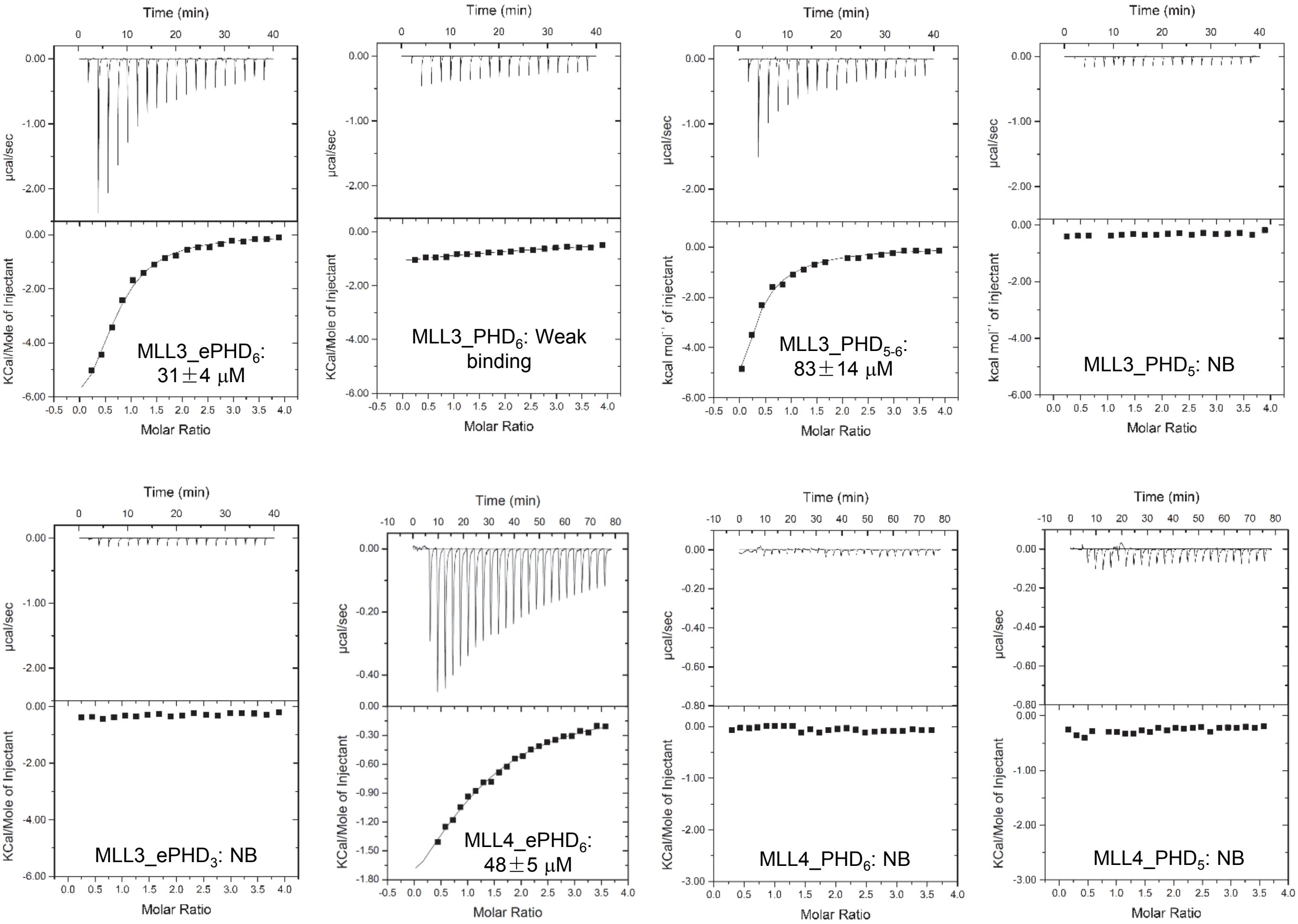

Supplementary Fig. 2 ITC titration curves for different MLL3 or MLL4 constructs to the histone H4 peptide $H4_{11\text{-}21}$. NB: no detectable binding. Related to Table 2.

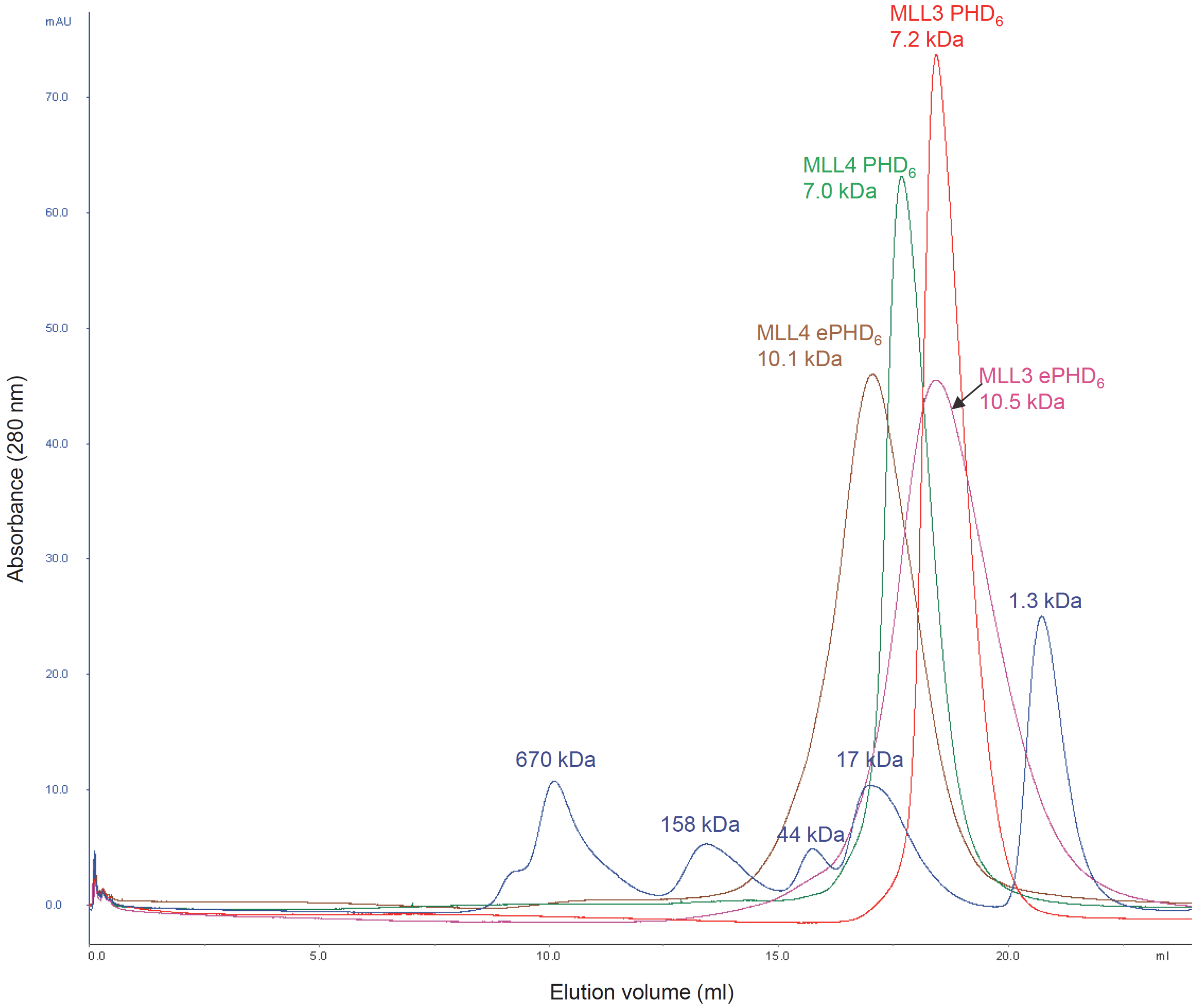

Supplementary Fig. 3 Superimposition of gel-filtration curves of PHD_6 and $ePHD_6$ domains of MLL3 and MLL4. Gel filtration chromatography of MLL3 $ePHD_6$ (red), PHD_6 (pink), MLL4 $ePHD_6$ (brown), PHD_6 (green) and protein molecular weight standards (blue, Bio-Rad). Samples were loaded to the Superdex200 10/300 GL (GE Healthcare) column in the buffer containing 20 mM Tris, pH 7.5, 150 mM NaCl, 50 µM $ZnCl_2$, and 1 mM DTT.

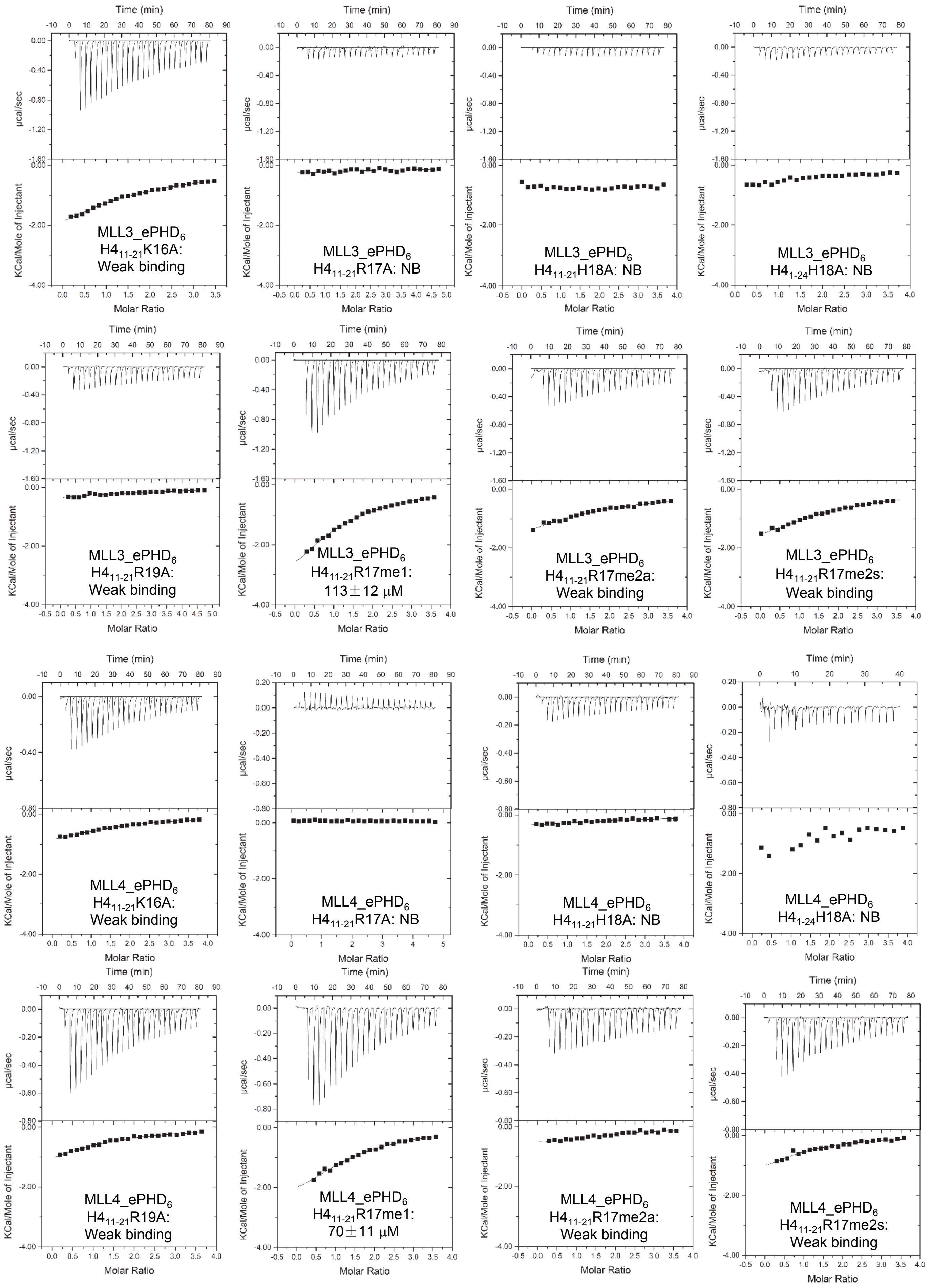

Supplementary Fig. 4 ITC titration curves for different histone H4 mutant peptides to the extended PHD$_6$ domains (ePHD$_6$) of MLL3 and MLL4. NB: no detectable binding. Related to Fig. 2h.

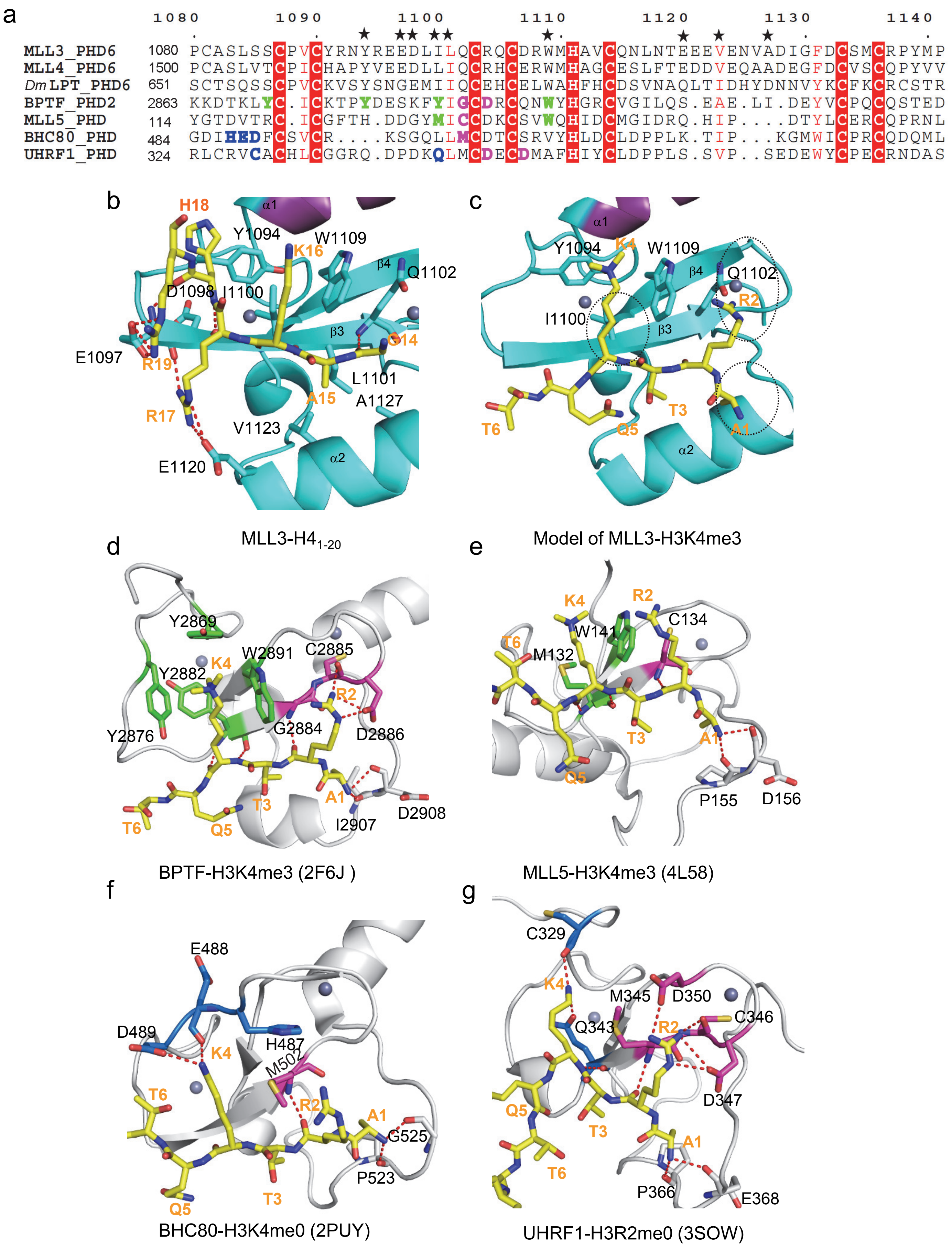

Supplementary Fig. 5 Structural comparison to other PHD domains. a Sequence alignment of different PHD domains. Residues involved in ligand binding are highlighted by black stars for MLL3 PHD6 and different colors for the other PHD domains. **b-g** Cartoon representation of different PHD domains. The bound histone peptides are shown in stick models.

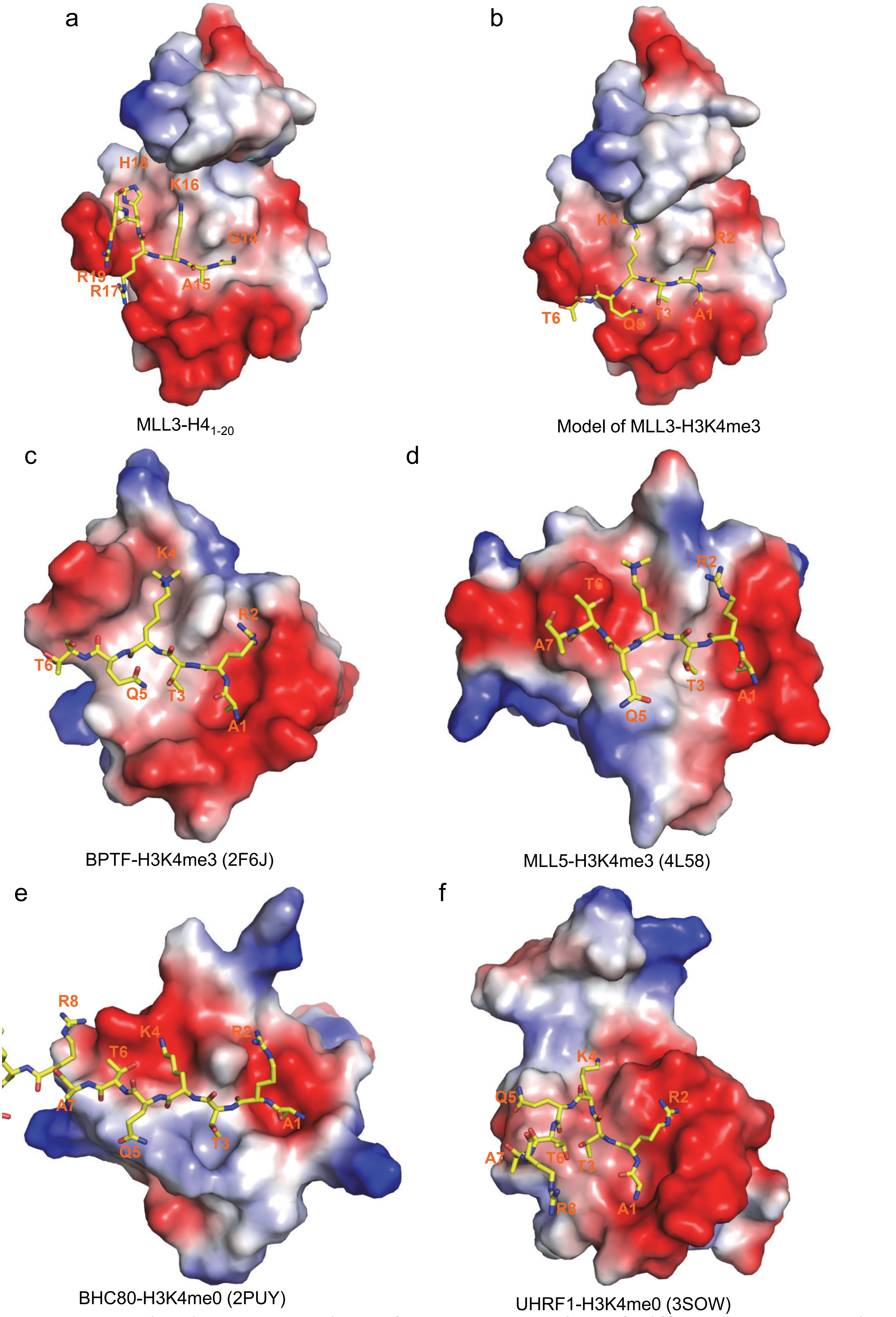

Supplementary Fig. 6: Electrostatic surface representation of different PHD domains. The bound Peptides are shown in stick models.

a

b

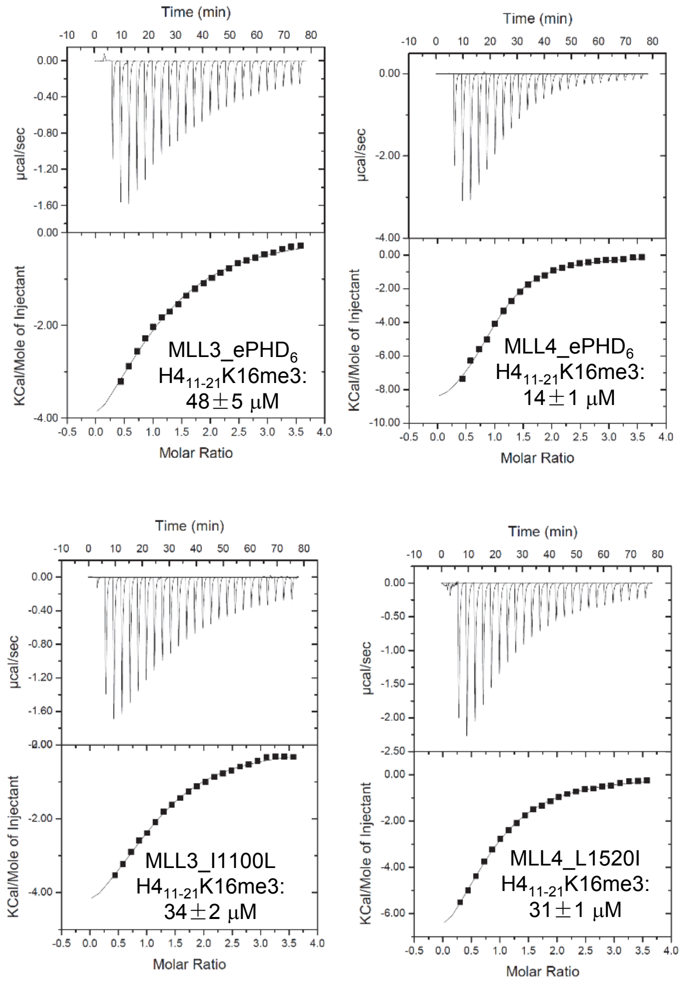

c

MLL3/MLL4 constructs	K_d (μM) H4$_{11\text{-}21}$K16me3
MLL3_(ePHD$_6$)	48 ± 5
MLL3_I1100L	34 ± 2
MLL4_(ePHD$_6$)	14 ± 1
MLL4_L1520I	31 ± 1

Supplementary Fig. 7 Trimethylation of H4K16 affects histone H4 binding to MLL3 and MLL4 differently. ITC titration curves (**a and b**) and affinity table (**c**) for the titration of H4$_{11\text{-}21}$K16me3 to the ePHD$_6$ domain of wt MLL3, MLL3_I1100L mutant, wt MLL4 and MLL4_L1520I mutant.

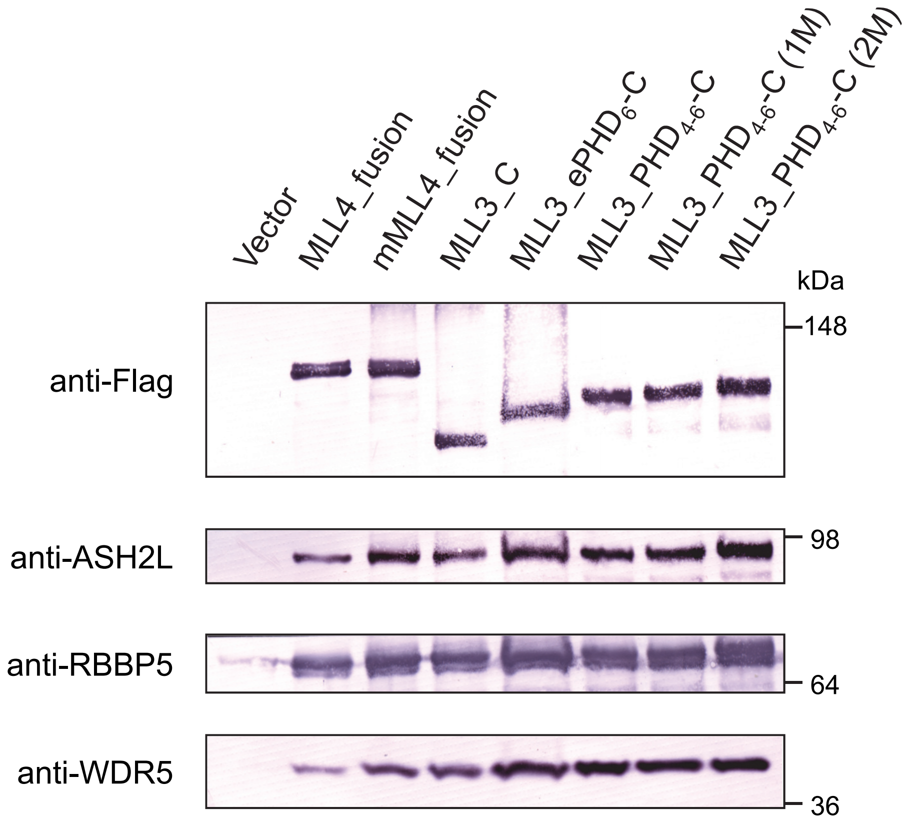

Supplementary Fig. 8 Western blot analysis of immunoprecipitation eluates of proteins from different flag-tagged MLL3/MLL4 constructs. The antibodies used for this assays are as following:

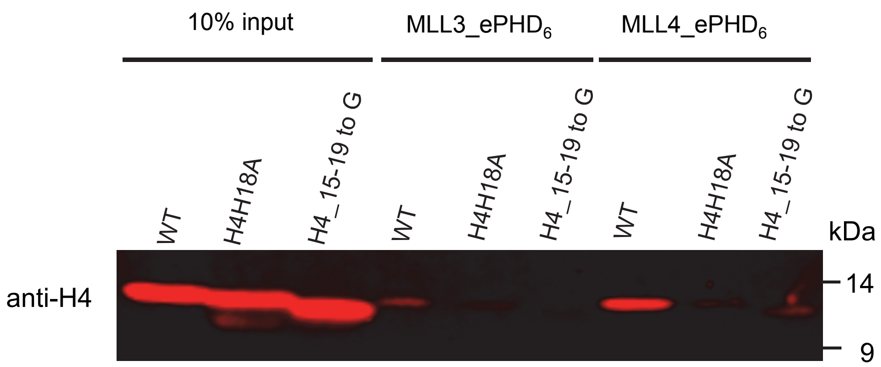

Supplementary Fig. 9 Nucleosome pull-down assay. Pull-down of wild type and mutant recombinant nucleosomes by GST-tagged ePHD$_6$ of MLL3/4, which was then detected by western blot analysis with anti-H4 specific antibody (Abcam, ab174628). The anti-H4 antibody is generated using the C-terminus of histone H4 as the antigen

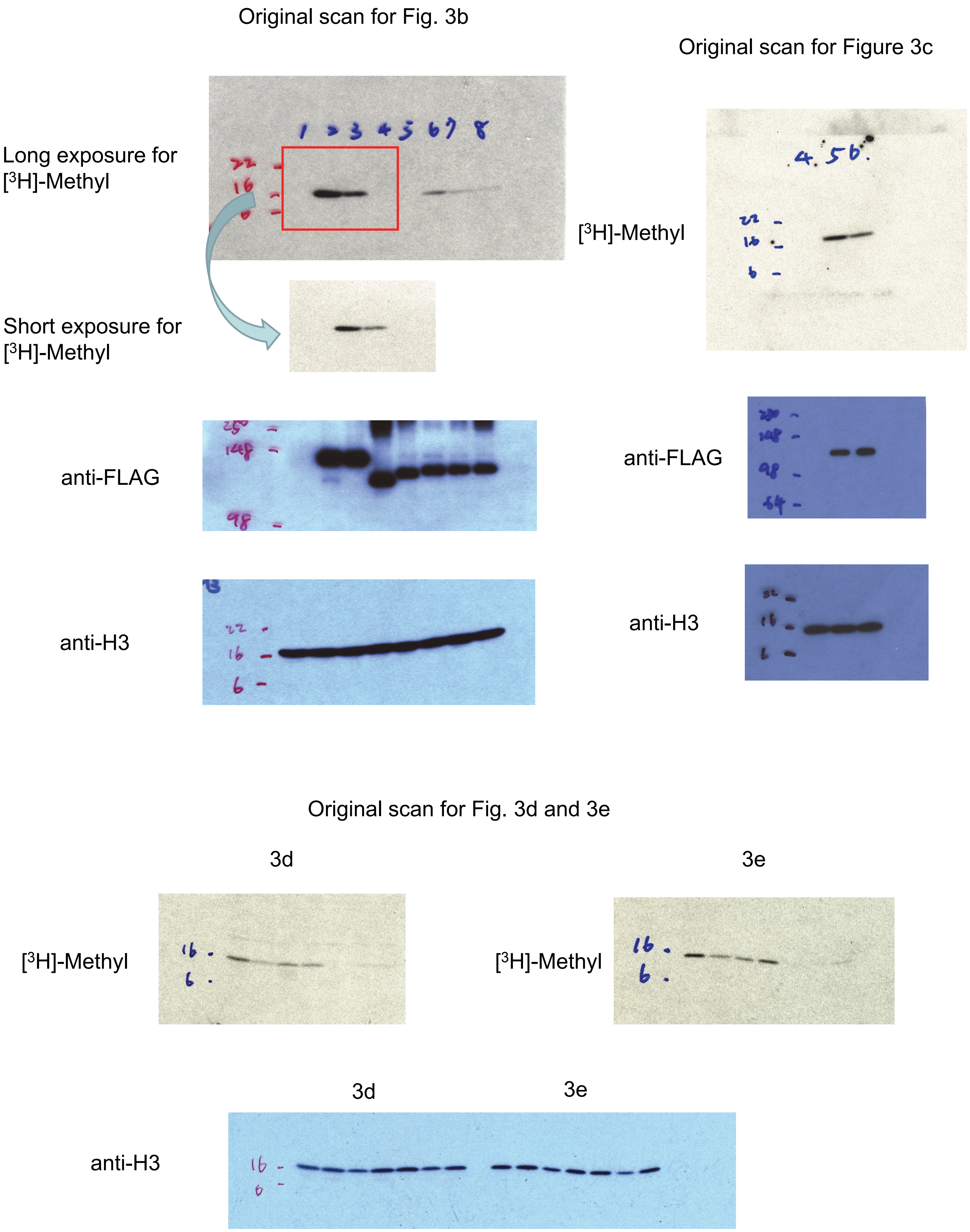

Supplementary Fig. 10 Original scan for Fig. 3

Original scan for Fig. 4a

anti-FLAG

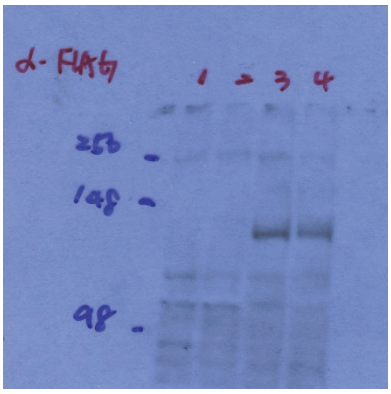

anti-β-Actin

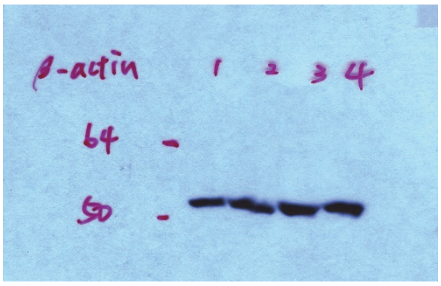

Supplementary Fig. 11 Original scan for Fig. 4a

Original scan for Supplementary Fig. 8

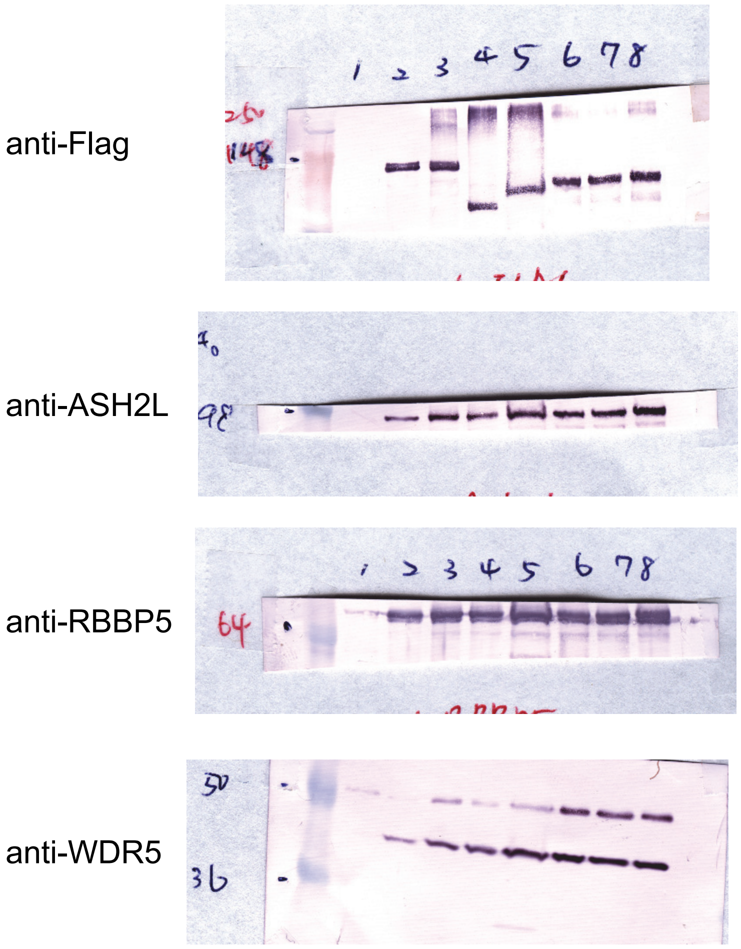

Supplementary Fig. 12 Original scan for Supplementary Fig. 8

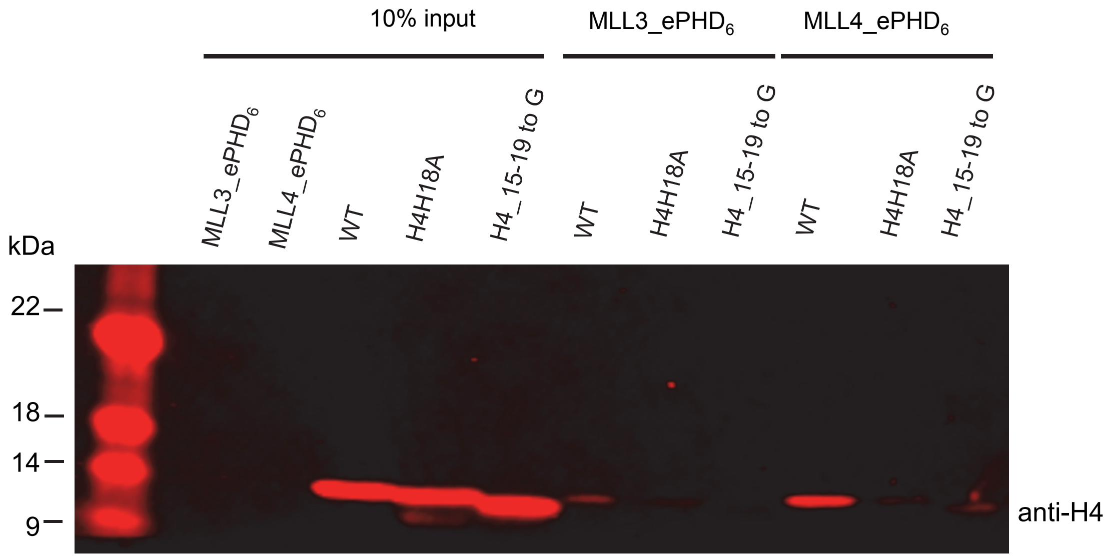

Supplementary Fig. 13 Original scan for Supplementary Fig. 9

Supplementary Table 1 Primer sequences

Protein fragments	Sequences
E coli expression constructs[a]	
MLL3_(ePHD$_6$)$_{1055-1144}$	5': gttccgcgtggtagtGTTTGGTGCAGACACTGTGG<br>3': caagcttcgtcatcaATTAGACGCAGGCATATAGGG
MLL3_(ePHD$_6$)$_{1075-1144}$	5': gttccgcgtggtagtTACACACAGTGCGCTCCTTG<br>3': caagcttcgtcatcaATTAGACGCAGGCATATAGGG
MLL3_(PHD$_6$)$_{1085-1144}$	5': gttccgcgtggtagtTCTTCCTGTCCAGTCTGCTATC<br>3': caagcttcgtcatcaATTAGACGCAGGCATATAGGG
MLL3_(PHD$_5$)$_{1009-1055}$	5': gttccgcgtggtagtGTGTGTGAGGCCTGTGGGAAG<br>3': caagcttcgtcatcaAACACACCATTTGCACTTCCAG
MLL3_(PHD$_{5-6}$)$_{1008-1144}$	5': gttccgcgtggtagtACTGTGTGTGAGGCCTGTGG<br>3': caagcttcgtcatcaATTAGACGCAGGCATATAGGG
MLL3_(ePHD$_3$)$_{436-525}$	5': gttccgcgtggtagtCGTATTTGCATTGAATGCGGTAC<br>3': caagcttcgtcatcaATCCATTTCCGCGCCCAGATG
MLL4_(ePHD$_6$)$_{1475-1564}$	5': gttccgcgtggtagtGTGTCCTGTATGCAGTGTGG<br>3': caagcttcgtcatcaCACAGGCTTTACCACGTAGG
MLL4_(ePHD$_6$)$_{1495-1564}$	5': gttccgcgtggtagtTACACACACTGTGGGCCCTG<br>3': caagcttcgtcatcaCACAGGCTTTACCACGTAGG
MLL4_(ePHD$_6$)$_{1505-1564}$	5': gttccgcgtggtagtGTGACCTGCCCTATCTGTCATG<br>3': caagcttcgtcatcaCACAGGCTTTACCACGTAGG
MLL4_(PHD$_5$)$_{1429-1475}$	5': gttccgcgtggtagtGTGTGTGAGGTGTGTGGCC<br>3': caagcttcgtcatcaCACACACCACTTGCACTTCC
Mutants[b]	
MLL3_ePHD$_6$ (E1097A)	5': CGAAACTATAGAGAAgctGATCTTATTCTGCAA<br>3': TTGCAGAATAAGATCagcTTCTCTATAGTTTCG
MLL3_ePHD$_6$ (W1109A)	5': AGACAATGTGATAGAgcgATGCATGCAGTTTGT<br>3': ACAAACTGCATGCATcgcTCTATCACATTGTCT
MLL3_ePHD$_6$ (E1120A)	5': CAGAACTTAAATACTgcgGAAGAAGTGGAAAAT<br>3': ATTTTCCACTTCTTCcgcAGTATTTAAGTTCTG
MLL4 fusion-2M<br>MLL4 fusion (W1529A)	 5': CGCCACTGTGAACGGgcgATGCATGCAGGCTG<br>3': CAGCCTGCATGCATcgcCCGTTCACAGTGGCG
MLL4 fusion (E1540A)	5': GAGCCTCTTCACAgcgGACGATGTGGAGC<br>3': GCTCCACATCGTCcgcTGTGAAGAGGCTC
Nucleosome mutants<br>H4-1-5 to G	 5': ATGggaGGCggaGGAggaGGCGGAAAAGGC<br>3': GCCTTTTCCGCCtccTCCtccGCCtccCAT
H4-15-19 to G	5': GGGGGCggaggaggaggaggaAAGGTCTTG<br>3': CAAGACCTTtcctcctcctcctccGCCCCC

H4_R3A	5': ATGTCCGGCgcaGGAAAGGGCGGAAAAGGC<br>3': GCCTTTTCCGCCCTTTCCtgcGCCGGACAT
H4_R17A	5': AAAGGGGGCGCTAAGgccCACCGCAAGGTC<br>3': GACCTTGCGGTGggcCTTAGCGCCCCCTTT
H4_H18A	5': AAAGGGGGCGCTAAGCGCgcaCGCAAGGTC<br>3': GACCTTGCGtgcGCGCTTAGCGCCCCCTTT
Mammalian expression constructs[c]	
MLL3_C$_{3993-4911}$	5': ccatcgataGGTGATCGAGATACTCCTGAC<br>3': ggggtacctcGTTCATCCACTTCCGGCAGTTC
MLL3_(ePHD$_{6}$)$_{1055-1144}$	5': ataagaatgcggccgcgTACACACAGTGCGCTCCTTG<br>3': ggaattcgcATTAGACGCAGGCATATAGGG
MLL3_(ePHD4$_{-6}$)$_{939-1144}$	5': ataagaatgcggccgcgTCTATGCACAATACAGTTGTGTTG<br>3': ggaattcgcATTAGACGCAGGCATATAGGG
Quantitative RT-PCR	
HOXA1	5': TTCTCCGGCCCCATGG<br>3': GAGTGACCTGGTCCTGCGAA
HOXA2	5': TTTCATACCCGTAGGGCTCGG<br>3': CCCTGCTGGTAACTTCCAACAG
HOXA3	5': TGCTTTGTGTTTTGTCGAGACTC<br>3': CAACCCCTACCCCTGCCAAC
NANOG	5': TTTGTGGGCCTGAAGAAAACT<br>3': AGGGCTGTCCTGAATAAGCAG
NeuN	5': CCAAGCGGCTACACGTCTC<br>3': CGTCCCATTCAGCTTCTCCC

Note: a, the lower cases of these primers are the sequences used by T4 ligase-independent cloning; b, the lower cases of these primers are the mutation sites; c, the lower cases of these primers are the sequences associated with restriction enzyme sites, *Not* I at 5’ primer and *Eco*R I at 3’ primer for the PHD constructs and *Cla* I at 5’ primer and *Kpn* I at 3’ primer for the C terminal of MLL3 construct.
